# Combination of radiotherapy and targeted therapy for HER2-positive breast cancer brain metastases

**DOI:** 10.1186/s40001-022-00894-7

**Published:** 2023-01-16

**Authors:** Xiaojing Yang, Hanru Ren, Yi Xu, Xue Peng, Wenxi Yu, Zan Shen

**Affiliations:** 1 Department of Oncology, Shanghai Sixth People’s Hospital Affiliated to Shanghai Jiao Tong University School of Medicine, No. 600, Yishan Road, Shanghai, 200233 China; 2 Department of Orthopedics, Pudong Medical Center, Shanghai Pudong Hospital, Fudan University, Shanghai, China; 3 Department of Radiation Oncology, Shanghai Sixth People’s Hospital Affiliated to Shanghai Jiao Tong University School of Medicine, Shanghai, China; 4 Department of Breast Surgery, Shanghai Sixth People’s Hospital Affiliated to Shanghai Jiao Tong University School of Medicine, Shanghai, China

**Keywords:** Breast cancer, Human epidermal growth factor receptor 2, Brain metastasis, Radiation therapy, Targeted therapy

## Abstract

Radiotherapy and targeted therapy are essential treatments for patients with brain metastases from human epidermal growth factor receptor 2 (HER2)-positive breast cancer. However, the combination of radiotherapy and targeted therapy still needs to be investigated, and neurotoxicity induced by radiotherapy for brain metastases has also become an important issue of clinical concern. It remained unclear how to achieve the balance of efficacy and toxicity with the application of new radiotherapy techniques and new targeted therapy drugs. This article reviews the benefits and potential risk of combining radiotherapy and targeted therapy for HER2-positive breast cancer with brain metastases.

## Background

Breast cancer is one of the most common malignant tumors among women in China. It is one of the tumors with the highest incidence of brain metastases, second only to lung cancer, accounting for 10–16% of brain metastases [1]. Brain metastasis is a significant factor in the decline of overall survival (OS) and degradation of the quality of life in breast cancer patients. Studies have confirmed that 20% of breast cancer patients die of brain metastases [2]. Since most drugs cannot pass through the blood–brain barrier (BBB), chemotherapy alone has a limited effect [3]; additionally, brain metastases are mostly multiple foci, which makes them difficult to be controlled by surgery [4]. Brain radiotherapy is the most commonly used and effective palliative treatment for brain metastases [5]. Brain radiotherapy includes whole-brain radiotherapy (WBRT), WBRT + local supplementation, WBRT + stereotactic radiosurgery (SRS)/stereotactic radiotherapy (SRT), and SRS/SRT [6]. However, there are not many studies on the evaluation of brain radiotherapy effects, especially the improvement of systemic treatment in recent years. Whether the pattern of radiotherapy has changed is still unclear.

About 20–25% of breast cancer patients have human epidermal growth factor receptor 2 (HER2) gene overexpression [7]. Anti-HER2 therapy is a crucial therapeutic strategy to improve the survival prognosis of this subgroup of patients. Under the background of molecular-typing-guided individualized comprehensive treatment, the incidence of brain metastases has also increased year by year with the prolongation of survival of HER2-positive breast cancer patients [8]. HER2-positive breast cancer brain metastases (BCBM) are characterized by multiple small lesions. They are often accompanied by leptomeningeal metastases, and those with meningeal metastases tend to have a poor prognosis [9]. Radiotherapy is the primary strategy for local treatment of patients with BCBM, and the combination of radiotherapy and targeted therapy can bring survival benefits to HER2-positive patients. Recent scientific evidence is demonstrating radiotherapy is capable of improving the quality of life and curative effect of metastases in metastatic prostate cancer [10]. Therefore, objective evaluation of the efficacy and adverse reactions of the combination of radiotherapy and targeted therapy is of great value in optimizing the comprehensive treatment of HER2-positive BCBM patients.

## Characteristics of HER2-positive BCBM

In 2000, Perou et al. first proposed the concept of breast cancer molecular typing based on gene chip technology [11]. Then, molecular typing based on immunohistochemistry was further suggested in the St. Gallen International Consensus on Breast Cancer in 2011 [12]. The proposal and refinement of this concept have promoted the development of clinical research on individualized systemic therapy for breast cancer. The guiding value of molecular typing for systemic treatment strategies is now well recognized. The incidence of BCBM is also related to molecular typing, and HER2-positive metastatic breast cancer is the subtype in which brain metastasis occurs with the highest risk [8]. The HERA study followed up early breast cancer patients receiving anti-HER2 therapy for 11 years and found that the rate of central nervous system (CNS) metastases was 2.1% [13]. At the same time, with the progression of the disease course, the incidence of brain metastases in patients with metastatic HER2-positive breast cancer also increased, and the proportion was as high as 37.2% in the registHER study [14].

This finding also led to the reconsideration on the risk of brain metastases under anti-HER2 therapy for HER2-positive breast cancer. The use of anti-HER2 therapy drugs such as trastuzumab may contribute to the development of brain metastases. Park et al. [15] analyzed single-center data and found that the occurrence rate of HER2-positive BCBM was 37.8% in the pre-trastuzumab era, and decreased to 25% since the clinical application of trastuzumab (*P* = 0.028). In addition, the median time to the diagnosis of brain metastases improved from 10 to 15 months in patients receiving trastuzumab treatment [15]. This result suggests that adjuvant anti-HER2 therapy delays the overall development of brain metastases through effective extracranial control.

In addition, the Breast Cancer Brain Metastasis Graded Prognostic Assessment (Breast-GPA) indicated that the median score of patients with scores ranging from 0 to 1.0, 1.5 to 2.0, 2.5 to 3.0, and 3.5 to 4.0 The survival times were 3.4, 7.7, 15.1 and 25.3 months, respectively. The HER2-positive single index score was 1.5 points [16]. This reflects the vital role of HER2 expression in the prognosis of patients with brain metastases, and the prognosis of patients with HER2-positive brain metastases seems to be better. A retrospective study included 112 BCBM patients, of whom the median survival time of the whole group was 14.4 months, and the median survival time of HER2-positive patients was 231 months [17]. A retrospective analysis of 66 HER2-positive BCBM patients by Pessina et al. showed that more than 50% of patients who received anti-HER2 therapy after brain metastases had a 3-year OS of 49%, while those who did not receive anti-HER2 treatment had a 3-year survival rate was only 33% (*P* = 0.03) [18]. This suggests that under the premise of effective anti-HER2 therapy, the prognosis of HER2-positive BCBM patients is relatively good.

## Intracranial radiotherapy combined with targeted therapy for HER2-positive BCBM

### Selection of radiotherapy techniques for brain metastases

**Fig. 1:**
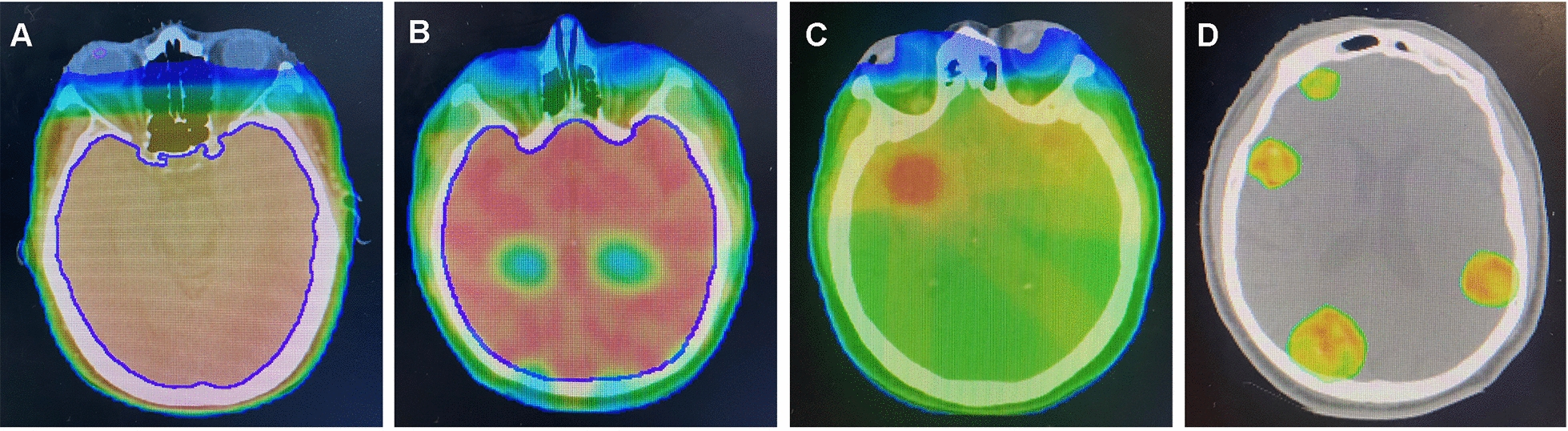
Evolution of brain radiotherapy: **A** whole-brain radiotherapy; **B** hippocampal-avoidance technique; **C** whole-brain radiotherapy and simultaneous integrated boost technique; **D** multiple-site stereotactic radiotherapy

Radiation therapy for brain metastases is available in a variety of ways (Fig. 1):

- WBRT is the earliest non-surgical treatment for brain metastases, which can relieve neurological symptoms and improve local control. However, studies have shown that whole-brain radiotherapy can impair the cognitive function of patients [6]. For larger brain tumors, increasing the total radiation dose can effectively improve the efficacy of radiotherapy. Compared with whole-brain radiotherapy, WBRT combined with local tumor intensity modulated radiation therapy (IMRT) is currently the primary treatment option for cancer brain metastases [19].
- Stereotactic radiotherapy (SRT) uses modern imaging technology to correctly find the target area, accurately delineate the target area through stereotaxic and verification technology, and drive the particular ionizing radiation device through computer control to destroy the target area with high dose. Minimizing radiotherapy’s side effects and radiation damage is a modern radiotherapy process characterized [20]. Compared with traditional radiotherapy, SRT has the advantages of a higher degree of precision, a lower radiation dose to organs at risk (OAR), and a higher radiation dose to tumor target areas [21]. SRT adopts multiple fractionation treatment methods, which is more in line with the requirements of radiobiology. The cells with sub-lethal damage to normal tissue after irradiation are almost entirely recovered within the interval between fractionated treatments. Tumor-hypoxic cells reoxidize, and cells in the G0 phase enter the radiosensitive phase. This may lead to better control for malignant tumors without causing serious damage to normal tissues [22]. The characteristics of dose distribution are: the composite dose distribution of multi-line beams is concentrated after spatial beam focusing, and the dose gradient around the target area varies considerably; the dose distribution in and near the target area is uneven; the dose around the target area is low [23]. Therefore, SRT is often used in the treatment of brain tumors. SRT can control the lesion site qualitatively and quantitatively and deliver the dose with precise local control. Therefore, it can improve the OS of patients, and reduce the incidence of adverse reactions. Many scholars believe that it has broad prospects in treating multiple brain metastases.
- SRS has the advantages of accurate positioning, concentrated dose, relatively minor damage, and short treatment time [24]. SRS has a good effect on isolated brain metastases, and its indications include [25–27]: (1) the diameter of single initial metastases was less than 5 cm. (2) The number of metastases for initial treatment should not exceed four. (3) Salvage therapy after WBRT failure. (4) Adjuvant therapy after resection of intracranial metastases. (5) SRS may be reconsidered in patients who have previously received SRS with a duration of response for more than 6 months, and the tumor is considered recurrent rather than necrotic on imaging. (6) Local augmentation of localized meningeal metastases based on WBRT treatment.
- CyberKnife (CK) is a small linear accelerator installed on a robotic arm with 6 degrees of freedom. There are 160 nodes on spherical surfaces of different radii, which can form 1980 incident directions, thus achieving relatively adequate coverage of the target area and a higher dose gradient at the edge of the target area [28]. The research of Zhou et al. regarded CK as a newly developed SRT technology, using non-isocentric and non-coplanar circular field technology to treat brain tumors [29]. Combining multiple incident directions and the application of reverse planning can achieve high target conformity and minimize the damage to surrounding normal tissues caused by high-dose fractionation.

For early and timely treatment of brain metastases, radiotherapy is a more critical part, and there are various methods to choose from. SRT has the advantages of precise target positioning, concentrated dose, and high target conformity. As a new type of SRT technology, CyberKnife has excellent benefits for isolated lesions. Still, because of its huge expense, it is difficult to be widely promoted. According to the expert group, SRT combined with WBRT is recommended for patients with high-risk intracranial metastases [30].

### Selection of targeted drugs

HER2 upregulation enhances the role of growth signals in the extracellular environment, promoting cell survival and proliferation through various downstream effects. Drugs targeting HER2 can block this downstream effect and improve outcomes in patients with HER2-positive breast cancer, but also increase the incidence of brain metastases [31]. The main HER2-targeted drugs include monoclonal antibodies, antibody–drug conjugates (ADCs), and tyrosine kinase inhibitors (TKIs) [32]. Macromolecular monoclonal antibodies can bind to the extracellular part of the HER2 molecule, and small-molecule TKIs can block the signal transduction of the intracellular segment of the HER2 molecule to exert antitumor effects. ADCs can be coupled with cytotoxic drugs based on HER2-targeting monoclonal antibodies, thereby further killing tumor cells [33]. Several clinical trials have explored the efficacy of HER2-targeting mAbs, ADCs, and TKIs in HER2-positive BCBM.

#### Monoclonal antibodies

##### Trastuzumab

One study demonstrated a 1:420 trastuzumab-to-plasma concentration ratio in the cerebrospinal fluid of patients with BCBM who did not receive radiation therapy. Radiation therapy or surgery can increase this ratio value, but the effect is still limited. In leptomeningeal metastases, intrathecal injection of trastuzumab allows the drug to bypass the BBB and reach therapeutic concentrations in the cerebrospinal fluid, thereby prolonging patient survival [34]. The study by Park et al. found that trastuzumab significantly improved BCBM outcomes. It is mainly related to its ability to control extracranial lesions effectively in the long term [15]. In addition, a preclinical study by Kodack et al. found that the combination of trastuzumab and the small-molecule TKI lapatinib with anti-vascular drugs can delay the progression of intracranial lesions [35]. Phase I NCT00543504 trial further confirmed the effectiveness of the therapy [36]. Among the ten BCBM patients, six had progression-free survival (PFS) of more than 6 months, of which one had a PFS of more than 12 months, and no adverse reactions related to brain metastasis occurred. In the TUXEDO-1 trial, trastuzumab showed a high intracranial response rate in patients with HER2-positive BCBM [37].

##### Pertuzumab

Pertuzumab combining with trastuzumab and taxanes is currently the standard first-line treatment for HER2-positive advanced breast cancer. The phase III CLEOPATRA trial showed that compared with trastuzumab and docetaxel alone, trastuzumab and pertuzumab in combination with docetaxel resulted in prognosis improvement in patients with metastatic breast cancer [38]. Both PFS and OS were significantly prolonged. Swain et al. found that compared with the trastuzumab alone group, the time to occurrence of brain metastases was longer in the trastuzumab plus pertuzumab group (15.0 months VS 11.9 months, *P* = 0.005) [39]. Given that both trastuzumab and pertuzumab can bring survival benefits to patients with BCBM, it is necessary to dive further into the effect of macromolecular mAbs on BCBM.

#### ADCs

##### Trastuzumab emtansine (T-DM1)

T-DM1 is the current standard second-line treatment for HER2-positive advanced breast cancer. T-DM1 consists of trastuzumab and the microtubule inhibitor emtansine. The phase III EMILIA trial included 95 treated and asymptomatic BCBM patients, of whom 45 received T-DM1 therapy. Compared with lapatinib combined with capecitabine, T-DM1 significantly prolonged OS in BCBM patients (26.8 months VS 12.9 months, *P* = 0.008) [40]. A subgroup analysis of the phase IIIb KAMILLA trial showed that among 126 patients with stable brain metastases at baseline, 84 patients had brain metastases shrinking during T-DM1 treatment, indicating that T-DM1 is effective in the treatment of BCBM [41]. The KATE2 trial showed that T-DM1 combined with atezolizumab brought a survival benefit to patients with programmed death-ligand 1 (PD-L1)-positive metastatic breast cancer. Still, BCBM patients were not included, so the role of this therapy in BCBM requires further study [42].

##### DS-8201

DS-8201 consists of trastuzumab and the topoisomerase I inhibitor deruxtecan. The phase II DESTINY-Breast01 trial explored its effectiveness as a post-line therapy. For 184 patients with metastatic breast cancer who once received T-DM1 in the past, DS-8201 was used for treatment, the median PFS was 16.4 months, and the objective response rate (ORR) assessed by the independent review committee was 60.9%. Of these, 24 were treated patients with BCBM who had no symptoms associated with brain metastasis and had a median PFS of 18.1 months. It is worth noting that the incidence rate of interstitial lung disease is higher after DS-8201 treatment, and pulmonary symptoms should be monitored during treatment [43, 44].

##### SYD985

SYD985, consisting of trastuzumab and the alkylating agent duocarmycin, has been granted by the U.S. Food and Drug Administration (FDA). The phase I NCT02277717 trial dose-expansion cohort enrolled 146 patients with metastatic breast cancer, including 8 patients with BCBM, and the results showed that SYD985 was safe and effective [45]. However, there is no published data on the treatment of brain metastases with SYD985, so the role of SYD985 in BCBM needs further study.

#### Small molecule TKI drugs

##### Lapatinib

Lapatinib is a small-molecule TKI with two targets epidermal growth factor receptor (EGFR) and HER2. The phase II LANDSCAPE trial showed that lapatinib combined with capecitabine was effective in BCBM who had not received radiation therapy before, and the ORR was 65.9% for the CNS. In patients with HER2-positive BCBM, lapatinib combined with capecitabine can achieve a PFS of 8.3 months, and subsequent radiation therapy can achieve an OS of up to 17 months [46]. In a phase I clinical trial, 35 HER2-positive BCBM patients received lapatinib combined with whole-brain radiotherapy, and the ORR was 79% [47]. Lu et al. developed a dual-targeted micellar delivery system loaded with paclitaxel (PTX) and lapatinib for the combined treatment of BCBM. Prolonged lifespans with brain metastases have been demonstrated in a mouse model [48]. This study provides a promising novel approach for treating BCBM.

##### Neratinib

Neratinib irreversibly inhibits the activity of HER1, HER2, and HER4. The NEfERT-T trial compared neratinib plus paclitaxel with trastuzumab plus paclitaxel in metastatic breast cancer. The results show that the efficacy of the two is similar, but neratinib combined with paclitaxel can delay and reduce the progression of brain metastases [49]. The phase II TBCRC022 trial further demonstrated the efficacy of neratinib in HER2-positive BCBM. Over 90% of patients had CNS progression at enrollment, and the ORR for the CNS was 49% and 33% in lapatinib-naïve and lapatinib-naïve patients, respectively. Median PFS were 5.5 months and 3.1 months, respectively [50]. The phase III NALA study found that neratinib plus capecitabine significantly prolonged PFS compared with lapatinib in patients with metastatic breast cancer. The main reason is the reduction of patients with symptoms of brain metastases requiring intervention [51].

##### Tucatinib

Tucatinib is a reversible TKI that is highly selective for HER2 and has shown promising intracranial activity in both preclinical and clinical studies. Compared with placebo plus trastuzumab and capecitabine, tucatinib plus trastuzumab and capecitabine significantly reduced the risk of disease progression and death. Among the 291 patients with BCBM, 1-year PFS was 24.9% in the tucatinib group and 0 in the placebo group, and the median PFS was 7.6 months and 5.4 months, respectively [52]. Based on the results, tucatinib was granted priority approval by the FDA. This regimen is indicated for treating patients with locally advanced unresectable or metastatic HER2-positive breast cancer, including BCBM, which have progressed after undergoing three or more HER2-targeted therapies [53]. In addition, a phase Ib clinical trial found that tucatinib combined with T-DM1 was also influential in BCBM with fewer adverse reactions [54].

##### Pyrotinib

Pyrotinib is a broad-spectrum anti-HER1, HER2, and HER4 intracellular small-molecule TKI independently developed in China [55]. The phase III clinical trial data in HER2-positive metastatic breast cancer showed that PFS was significantly prolonged in the pyrotinib group compared with the placebo group (11.1 months VS 4.1 months). For patients with no brain metastases at baseline, the pyrotinib group had a lower rate of new brain metastases than the placebo group (1.2% VS 3.6%). The time to onset of new brain metastases was longer (397.5 days VS 132.0 days). For patients with untreated brain metastases at baseline, the pyrotinib group had a lower proportion of patients with brain metastases (73.3% VS 87.5%) and a longer time to brain metastases (168.0 d VS 127.0 d) [56]. Tian et al. evaluated the efficacy of pyrotinib in patients with HER2-positive BCBM undergoing WBRT. Twenty patients were divided in a 1:1 ratio into the pyrotinib plus capecitabine group and the capecitabine-only group. Oral pyrotinib combined with radiotherapy significantly improved OS, PFS, and duration of response in patients with HER2-positive BCBM without additional adverse events [57]. Multiple real-world data show that pyrotinib improves outcomes in patients with HER2-positive BCBM [58–60].

### The possible mechanism of radiotherapy combined with HER2-targeted therapy

**Fig. 2:**
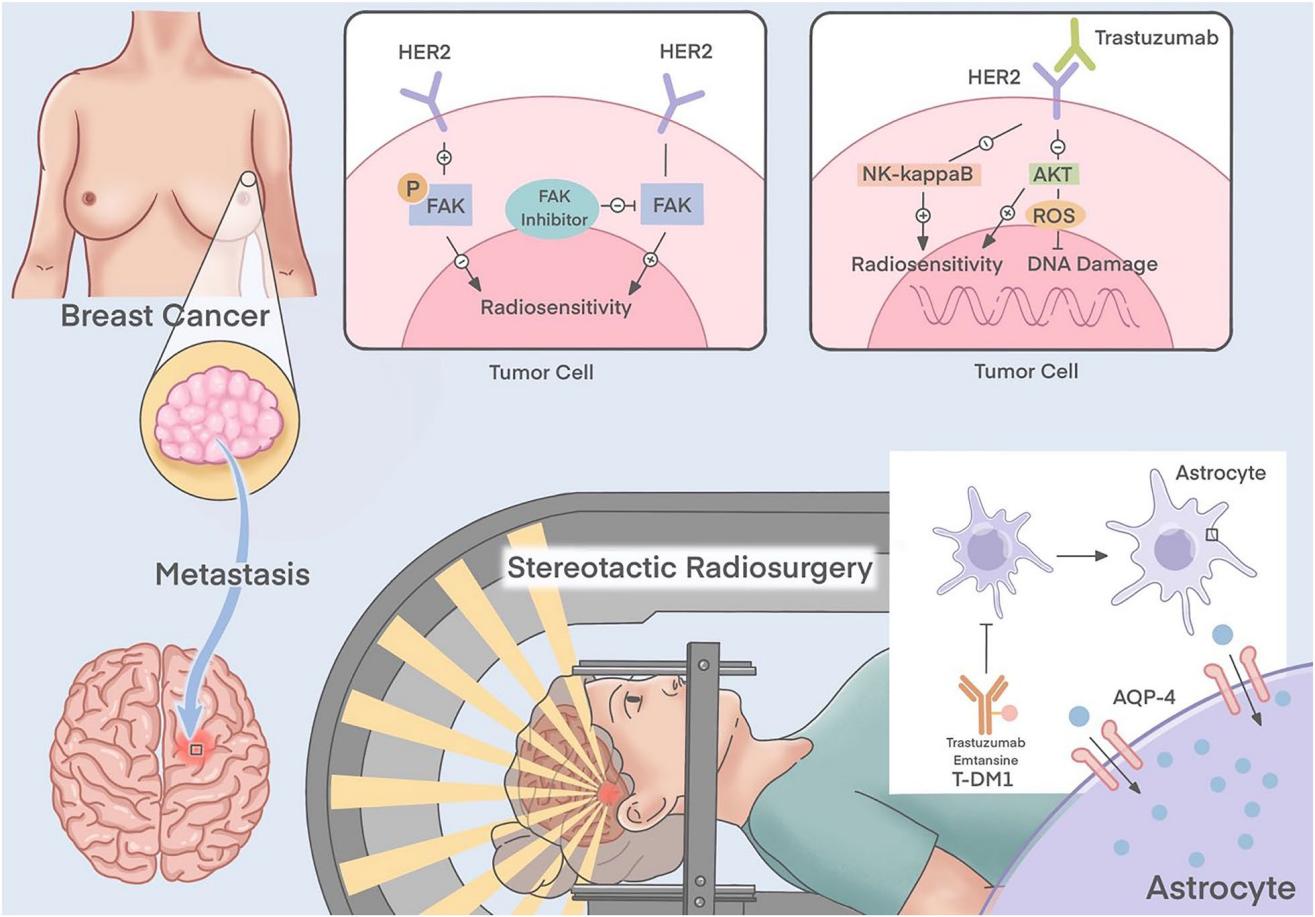
Possible mechanism of radiotherapy combined with HER2-targeted therapy

Trastuzumab was administered concurrently with radiotherapy and demonstrated HER2 radiosensitization in mouse models [61]. According to Pietras et al., trastuzumab-induced radiosensitization alters cellular DNA repair and cell cycle processes mediated by phosphatidylinositol 3-kinase (PI3K) and Akt [62]. Intracellular signaling cascades mediated by PI3K and Akt are the origin of many biological processes, including cell growth, angiogenesis, long-range migration, and cellular uptake of glucose [63]. Trastuzumab also appears to inhibit Akt and NF-kappaB-induced radioresistance [64]. There is growing evidence that HER2 is involved in breast cancer cells' response to radiation. A related schematic is shown in Fig. 2. By establishing mouse models of cardiac injury with radiotherapy mono-treatment using trastuzumab, and the combination therapy, Yi et al. analyzed whether these two treatments have synergistic effects. They believe that trastuzumab inhibits Akt phosphorylation, thereby promoting intracellular DNA damage. This exacerbates the damaging effects of radiation on cardiomyocytes in vitro [65]. Hou et al. showed that HER2 promotes the phosphorylation of focal adhesion kinase (Fak), thereby upregulating the expression of proteins associated with epithelial-mesenchymal transition (EMT). Inhibition of Fak activity with a Fak inhibitor (PF-562281) restored radiosensitivity in HER2-overexpressing cells. The results suggest that HER2 reduces the radiosensitivity of breast cancer by activating Fak. Fak may be a potential target for radiosensitization of HER2-overexpressing breast cancer cells [66].

There are also some preclinical studies of small-molecule TKIs combined with radiotherapy. The study demonstrated that ZD1839 and radiation therapy significantly inhibited tumor growth compared to performing either treatment alone. A quinazoline small-molecule TKI CI1033 in combination with radiation was examined for its effect in a panel of HER-overexpression breast cancer cell lines. Preclinical studies have shown that CI1033 is synergistic with ionizing radiation [67]. EGFR TKIs enhance the radioactivity of bladder cancer cells by synergistically blocking EGFR and HER2 [68]. As to esophageal cancer, pyrotinib sensitized HER2-positive esophageal cancer cells to radiotherapy by inhibiting HER2 phosphorylation, inducing G0/G1 phase arrest, thus reducing EMT and DNA repair [69].

SRS combined with T-DM1 was applied by Stumpf et al. in patients with HER2-positive brain metastases. T-DM1 targets reactive astrocytes. It increases radiosensitivity by increasing radiation-induced cytotoxicity and astrocyte swelling by upregulating Aquaporin-4 (Aqp4) [70].

### Efficacy of intracranial radiotherapy combined with targeted therapy on intracranial lesions

In the past, it was believed that most chemotherapeutic drugs and targeted therapy drugs are difficult to penetrate the BBB, so the therapeutic effect of systemic therapy on brain metastases is limited [71]. Radiotherapy is the standard treatment for patients with brain metastases. However, HER2-positive brain metastases patients had increased trastuzumab concentrations in the cerebrospinal fluid after WBRT. The blood-cerebrospinal fluid concentration ratio of trastuzumab before and after WBRT was 420:1 and 76:1, respectively. These data theoretically demonstrate that HER2-positive BCBM patients can benefit from subsequent anti-HER2 therapy based on WBRT [72].

Chargari et al. took the lead in analyzing WBRT and concurrent or continuous trastuzumab in 31 HER2-positive BCBM patients in 2011. The objective effective rate was 74.2%, the median survival time was 18 months, and the median intracranial disease control time was 10.5 months [73]. Since then, more and more studies have confirmed that WBRT and anti-HER2 therapy can achieve a median OS of 12.8 to 34 months in HER2-positive patients, while the median OS of patients receiving WBRT alone is ≤ 10 months [74–76]. Anti-HER2-treated patients with brain metastases have improved outcomes. In addition, T-DM1 combined with radiation, high-energy focused ultrasound, macitentan, or tucatinib have all achieved good results in BCBM [77]. However, Stumpf et al. found that T-DM1 combined with SRS may increase the incidence of radionecrosis. Given the small number of patients in this study, more extensive prospective studies are needed to evaluate the safety, maximum tolerated dose, and administration time of T-DM1 combined with SRS [70]. Guidelines for treating HER2-positive BCBM were released at the 2018 American Society of Clinical Oncology (ASCO) annual meeting. Although radiotherapy remains the standard option for asymptomatic, low-burden brain metastases that have not been treated with radiotherapy, lapatinib and capecitabine are also considerable options before radiotherapy [78].

Tian et al. showed that pyrotinib could enhance the radiosensitivity of cultured HER2-positive breast cancer cell lines. Pyrotinib is a potent drug that enhances in vivo tumor radiosensitivity with HER2-positive BCBM. Pyrotinib combined with radiotherapy can significantly improve the prognosis of patients with HER2-positive BCBM [57]. Real-world, multicenter data show that radiotherapy and pyrotinib can statistically improve OS in patients with HER2-positive metastatic breast cancer and brain metastases [58].

### Curative effect of targeted therapy on extracranial lesions

Why intracranial radiotherapy combined with targeted therapy improves OS compared with radiotherapy alone? The reason is that the use of anti-HER2 therapy cannot only effectively control intracranial lesions but also extracranial metastases. The proportions of HER2-positive BCBM patients who died of extracranial disease progression with or without trastuzumab were 11.9% and 37.1% (*P* = 0.014), respectively [15]. A retrospective study by Zhang et al. showed that the median OS of patients with HER2-positive brain metastases with and without anti-HER2 therapy after WBRT were 34 months and 8 months, respectively (*P* = 0.001). Meanwhile, the death rates from extracranial disease progression in patients with and without anti-HER2 therapy were 20.0% and 73.3%, respectively [75]. This shows that intracranial radiotherapy combined with anti-HER2 treatment can effectively control intracranial and extracranial metastases in patients with brain metastases.

### Time sequence and selection of targeted therapy and intracranial radiotherapy

Based on the survival benefit of anti-HER2 therapy in BCBM and the cognitive impairment caused by intracranial radiotherapy, more and more scholars are exploring the timing selection of targeted therapy and intracranial radiotherapy after brain metastases. The LANDSCAPE study explores the efficacy of lapatinib combined with capecitabine as first-line treatment in untreated HER2-positive BCBM patients [46]. The ORR of the combination therapy was as high as 65.9%. Due to the lack of higher-level data, there is no generally accepted recommendation for a comprehensive treatment strategy for patients with HELL2-positive breast cancer. Still, it suggests that in some patients with low metastatic burden and effective antitumor drug treatment, close follow-up can be performed. Intracranial radiotherapy can be safely delayed under the premise.

Stavrou et al. compiled a schematic diagram to guide us on which treatment method to choose based on the prognosis, tumor size, and the number of BCBM patients [79]. Chan et al. developed an algorithm for the treatment of HER2-positive BCBM [80]. They recommend local therapy with surgery, SRS, or WBRT before systemic therapy. SRS is superior to WBRT in oligometastatic lesions. Anti-HER2 drugs combined with pertuzumab, trastuzumab, or taxane should be used as first-line therapy to control systemic symptoms. Systemic therapy can continue after local CNS therapy if isolated brain metastases worsen without extracranial progression. However, when CNS progression occurs with extracranial progression, anti-HER2 drugs should be switched according to the conventional HER2 treatment pathway. T-DM1 is the next-line treatment option after lapatinib-capecitabine combination therapy. Otherwise, the neratinib-capecitabine combination can be considered. Other treatments include new drugs such as tucatinib, T-DXd, and margetuximab, or other ones participating in clinical trials. The completed clinical trials of targeted drugs combined with radiotherapy in HER2-positive BCBM registered on ClinicalTrial are listed in Table 1. It is obvious that there are only a few such clinical trials, and the number of patients enrolled is small. We have to notice that although these clinical trials are all displayed as completed, most of the relevant results cannot be retrieved. Prospective clinical trials of BCBM with radiotherapy combined with targeted therapy are listed in Table 2. We look forward to the results of this part of the study.

**Table 1:** Completed clinical trials of targeted therapies combine with radiotherapy in HER2 + BCBM patients

NCT identifier	Drug	Radiation therapy	Others therapy	Number of participants	Study phase	Outcome
NCT 00470847	Lapatinib: orally, 750 mg twice on day one followed by 1000 mg, 1250 mg, or 1500 mg once daily	WBRT: 37.5 Gy, 15 fractions, began 1–8 days after starting lapatinib	Following WBRT, patients received trastuzumab intravenously 2 mg/kg weekly and lapatinib 1000 mg orally once daily	35	I	CNS-ORR was 79%
NCT 02135159	T-DM1: T-DM1 concomitant with RT (injection day 1, 22); T-DM1 during and after RT [sequential RT (start day 1) followed by T-DM1 (injection day 15, 36)]; RT before T-DM1 [sequential RT (start day 1) followed by T-DM1 (injection day 22, 43)]; T-DM1 before RT [T-DM1 (First injection day 1) followed by sequential and concomitant RT (start day 18), second injection T-DM1 day 22]	WBRT	Not applicable	36	I	Not applicable
NCT 01363986	Herceptin (trastuzumab): initial loading dose of 4 mg/kg i.v. infusion, followed by weekly doses of 2 mg/kg for up to 18 weeks	WBRT	Not applicable	3	II	Not applicable
NCT 02563925	Tremelimumab: administered every 28 days	WBRT or SRS	Durvalumab	28	Not applicable	Not applicable
NCT 01613482	Trastuzumab: weekly, 2 mg/kg of corporal weight	Cerebral prophylactic radiation: 24 Gy, 12 fractions of 2 Gy	Not applicable	13	III	Not applicable

*ORR* objective response rate, *T-DM1* trastuzumab emtansine, *WBRT* whole-brain radiotherapy, *SRS* stereotactic radiosurgery

**Table 2:** Ongoing clinical trials of targeted therapies combine with radiotherapy in HER2 + BCBM patients

NCT identifier	Study phase	Treatment	Primary end point
NCT04582968	Ib/II	Pyrotinib plus capecitabine combined with brain radiotherapy	Ib: safety and tolerability II: intracranial local tumor control rate
NCT04767828	IV	Pyrrolidine maleate and capecitabine combined with brain radiotherapy	Time of intracranial tumor progression
NCT05042791	II	Radiation combined with pyrotinib and capecitabine	CNS-ORR

*BCBM* breast cancer brain metastases, *CNS* central nervous system, *ORR* objective response rate

## Side effects of radiotherapy combined with targeted therapy

### Radiation brain necrosis (RN)

RN is one of the late and irreversible toxic reactions after intracranial radiotherapy, which can cause neurological dysfunction, sometimes even life-threatening. Since RN is a late poisonous reaction secondary to vascular endothelial injury, and its occurrence and development are based on the long-term survival of patients. Therefore, most reports focus on nasopharyngeal carcinoma and glioma after radiotherapy. In the past, the reported overall incidence of RN in BCBM was low, given the poor overall prognosis of patients with brain metastases, the incidence rate of primarily symptomatic brain necrosis was only 6–11% [81–83]. Under the background of comprehensive treatment, the survival of HER2-positive breast cancer has been significantly prolonged, and the long-term quality of life of BCBM patients has become a focus of attention.

In addition to trastuzumab, other targeted therapies, including macromolecular monoclonal antibodies, small-molecule TKIs, and cytotoxic drugs, have gradually clarified their benefits and therapeutic status in HER2-positive breast cancer brain metastases, respectively [40, 46, 84]. The combination of antitumor drugs may have a superimposed or sensitizing effect on radiation damage. With new anti-HER2 therapeutic drugs, reports of related radiation brain necrosis have gradually increased. Geraud et al. reported preliminary results of the combination of SRS and trastuzumab–maytansine conjugate in 12 patients with HER2-positive brain metastases. The ORR was 75%. In this study, the incidence of focal brain necrosis was 50% in the concurrent treatment group and 28.6% in the sequential treatment group [85]. Another case series report also showed that T-DM1 might lead to brain edema after SRS treatment [86]. In a study of 45 patients with HER2-positive BCBM treated with SRS combined with T-DM1 by Stumpf et al., RN was observed in 39.1% of patients treated with T-DM1, compared with 4.5% of patients who did not receive T-DM1, the incidence of RN increased 13.5-fold (*P* = 0.02) [70]. The strong correlation of SRS with the development of RNs after T-DM1 set the foundation of further prospective studies, in which changes in timing and dose of T-DM1 are controlled, in order to stratify the risk of RNs and mitigate toxicities. The experiments above are all small sample case studies. Despite this, the studies suggest that the occurrence of RN needs to be re-explored under the circumstances of re-course therapy, multi-line chemotherapy, and combined targeted therapy.

### Other side effects

The efficacy of lapatinib combined with WBRT in HER2-positive BCBM patients was evaluated in a phase I clinical trial. Dose-limiting toxicities, including grade III rash, diarrhea, hypoxia, and grade IV pulmonary embolism, occurred in 7 of 27 patients when lapatinib was administered at 1250 mg [47]. Other common side effects were diarrhea, fatigue, nausea, neutropenia, stomatitis, and abnormal liver function. Diarrhea is a common side effect, and the exact mechanism of HER1/HER2 TKI-induced diarrhea is still unclear. The complete inhibition of the HER family signaling pathway may cause intestinal complications [87]. No reports are showing that combined radiotherapy will aggravate these symptoms. In terms of radiation-induced toxicity, no expansion of pulmonary fibrosis was observed in a mouse model with concomitant trastuzumab radiotherapy [88].

## Outlook

Since the hippocampus plays a vital role in memory preservation, hippocampal-sparing WBRT or IMRT can significantly alleviate radiation-induced neurocognitive and improve patient’s quality of life by avoiding dose delivery to the hippocampus in WBRT [89, 90]. Memantine is a noncompetitive n-methyl-d-aspartate receptor antagonist for HER2-positive BCBM. It can help improve neurocognitive impairment caused by WBRT. Some studies have proposed using focused ultrasound (FUS), nanotechnology, and radiation-based remote effect therapy for treating HER2-positive BCBM. Among all the possible options, FUS plus microbubbles can temporarily destroy the tight junction of the blood–brain barrier to allow drugs to penetrate. Studies have confirmed that the combination of trastuzumab and FUS has anticancer activity on HER2-positive breast cancer in mouse models [90]. There are also studies showing that pulsed FUS pretreatment of brain tissue can promote multiple enhancements of transgene expression, improving the penetration and efficacy of gene carrier nanoparticles in the central nervous system [91]. These all show the therapeutic potential of this non-invasive technique for targeted drug delivery to the brain. Nanoparticles can be combined with many anticancer agents and have been successfully used as carriers to deliver therapeutics across the blood–brain barrier. Nanoparticles coated with tumor-penetrating peptides could be a promising treatment method for preventing brain metastases [90]. Distant effects of radiation refer to localized irradiation-induced tumor regression at non-irradiated remote tumor sites. This mechanism is believed to be able to fight the tumor by triggering a host immune response by immunogen cell death [89].

Other studies have shown that fatty acid-binding proteins hold promise as potential targets for the treatment of HER2-positive BCBM. It promotes the growth of HER2-positive breast cancer cells in the brain. Elevated expression is associated with lower patient survival and a higher incidence of brain metastases [92]. Sambade et al. reported the prognostic significance of four tissue biomarkers of gliosis, immune infiltration, hemorrhage, and necrosis in BCBM were associated with good prognosis in HER2-positive BCBM [93]. Understanding the brain microenvironment of BCBM can help improve prognosis and may reveal new therapeutic strategies for HER2-positive BCBM.

While the systemic treatment of metastatic breast cancer continues to improve, the treatment of brain metastases also requires continuous follow-up. Appropriate local and systemic treatment plans for BCBM patients should be formulated according to the anatomical features, molecular typing, and prognosis of brain metastases. Traditional treatments such as chemotherapy and endocrine therapy have specific effects on BCBM. Some new drugs such as ADCs, TKIs, CDK4/6 inhibitors, PARP inhibitors, and immune checkpoint inhibitors will also bring incredible survival benefits to BCBM patients. Currently, multiple clinical trials on BCBM are ongoing (Table 2), which will continue to bring new hope to BCBM patients.

## Conclusions

The combination of targeted therapy and radiotherapy is a new opportunity and challenge in the progress of comprehensive treatment of BCBM. For HER2-positive BCBM, the efficacy of WBRT or SRS combined with trastuzumab or other anti-HER2 therapy has been confirmed. The optimization of the timing of the combination therapy still needs further research and demonstration, and the toxic reactions such as cerebral radiation necrosis cannot be ignored. The safety of the combination therapy should be verified in a larger sample size. It is believed that the combination of radiotherapy and targeted therapy is increasingly safe and standardized based on more effective systemic treatment. Continuous effort is needed to keep this therapy optimized as clinical research progresses.

## Data Availability

Not applicable.
